# The necessity of a loading dose when prescribing intravenous colistin in critically ill patients with CRGNB-associated pneumonia: a multi-center observational study

**DOI:** 10.1186/s13054-022-03947-9

**Published:** 2022-04-04

**Authors:** Sheng-Huei Wang, Kuang-Yao Yang, Chau-Chyun Sheu, Wei-Cheng Chen, Ming-Cheng Chan, Jia-Yih Feng, Chia-Min Chen, Biing-Ru Wu, Zhe-Rong Zheng, Yu-Ching Chou, Chung-Kan Peng, the T.-CARE (Taiwan Critical Care, Infection) Group

**Affiliations:** 1 Division of Pulmonary and Critical Care Medicine, Department of Internal Medicine, Tri-Service General Hospital, National Defense Medical Center, No. 325, Section 2, Cheng-Gong Rd, Neihu 114, Taipei, Taiwan; 2 Graduate Institute of Medical Sciences, National Defense Medical Center, Taipei, Taiwan; 3 Department of Chest Medicine, Taipei Veterans General Hospital, Taipei, Taiwan; 4 Institute of Emergency and Critical Care Medicine, School of Medicine, National Yang Ming Chiao Tung University, Taipei, Taiwan; 5 Cancer Progression Research Center, National Yang Ming Chiao Tung University, Taipei, Taiwan; 6 Division of Pulmonary and Critical Care Medicine, Department of Internal Medicine, Kaohsiung Medical University Hospital, Kaohsiung Medical University, Kaohsiung, Taiwan; 7 Department of Internal Medicine, School of Medicine, College of Medicine, Kaohsiung Medical University, Kaohsiung, Taiwan; 8 Graduate Institute of Biomedical Sciences, China Medical University, Taichung, Taiwan; 9 Division of Pulmonary and Critical Care Medicine, Department of Internal Medicine, China Medical University Hospital, Taichung, Taiwan; 10 Department of Education, China Medical University Hospital, Taichung, Taiwan; 11 Division of Critical Care and Respiratory Therapy, Department of Internal Medicine, Taichung Veterans General Hospital, Taichung, Taiwan; 12 National Chung Hsing University, Taichung, Taiwan; 13 School of Medicine, National Yang Ming Chiao Tung University, Taipei, Taiwan; 14 Ph.D. Program in Translational Medicine, National Chung Hsing University, Taichung, Taiwan; 15 Rong Hsing Research Center for Translational Medicine, National Chung Hsing University, Taichung, Taiwan; 16 Division of Pulmonary Medicine, Department of Internal Medicine, Chung Shan Medical University Hospital, Taichung, Taiwan; 17 Division of Chest Medicine, Department of Internal Medicine, Taichung Veterans General Hospital, Taichung, Taiwan; 18 School of Public Health, National Defense Medical Center, Taipei, Taiwan

**Keywords:** Colistin, Nosocomial pneumonia, Loading dose, Nephrotoxicity, Carbapenem resistant

## Abstract

**Background:**

The importance or necessity of a loading dose when prescribing intravenous colistin has not been well established in clinical practice, and approximate one-third to half of patients with carbapenem-resistant gram-negative bacteria (CRGNB) infection did not receive the administration of a loading dose. The aim of this study is to investigate the efficacy and risk of acute kidney injury when prescribing intravenous colistin for critically ill patients with nosocomial pneumonia caused by CRGNB.

**Methods:**

This was a multicenter, retrospective study that recruited ICU-admitted patients who had CRGNB-associated nosocomial pneumonia and were treated with intravenous colistin. Then, we classified the patients into colistin loading dose (*N* = 85) and nonloading dose groups (*N* = 127). After propensity-score matching for important covariates, we compared the mortality rate, clinical outcome and microbiological eradication rates between the groups (*N* = 67).

**Results:**

The loading group had higher percentages of patients with favorable clinical outcomes (55.2% and 35.8%, *p* = 0.037) and microbiological eradication rates (50% and 27.3%, *p* = 0.042) at day 14 than the nonloading group. The mortality rates at days 7, 14 and 28 and overall in-hospital mortality were not different between the two groups, but the Kaplan–Meier analysis showed that the loading group had a longer survival time than the nonloading group. Furthermore, the loading group had a shorter length of hospital stay than the nonloading group (52 and 60, *p* = 0.037). Regarding nephrotoxicity, there was no significant difference in the risk of developing acute kidney injury between the groups.

**Conclusions:**

The administration of a loading dose is recommended when prescribing intravenous colistin for critically ill patients with nosocomial pneumonia caused by CRGNB.

**Supplementary Information:**

The online version contains supplementary material available at 10.1186/s13054-022-03947-9.

## Background

Hospital-acquired pneumonia (HAP) and ventilator-associated pneumonia (VAP) are common nosocomial infections and are associated with high morbidity and mortality worldwide [1, 2]. Carbapenem-resistant gram-negative bacteria (CRGNB) are among the major pathogens causing HAP and VAP, and the incidence of infection with CRGNB could be as high as 57.1% in patients with VAP [3]. The major CRGNB pathogens resulting in HAP and VAP include carbapenem-resistant *Acinetobacter baumannii* complex (CRAB), carbapenem-resistant Enterobacteriaceae (CRE), and carbapenem-resistant *Pseudomonas aeruginosa* (CRPA). The main treatment for CRGNB pneumonia involves tigecycline, carbapenem, sulbactam, ceftazidime/avibactam, and resurgence medicines, including fosfomycin and polymyxins [4, 5].

Colistin (polymyxin E) is one of the major therapeutic choices for CRGNB-associated pneumonia. It is intravenously administered in the prodrug form of colistin methanesulfonate/colistimethate sodium (CMS), which is less nephrotoxic than colistin and is hydrolyzed to the active form in the plasma [6]. Colistin displays bactericidal activity against CRGNB via mechanisms involving the disruption of the outer membrane and the neutralization of lipopolysaccharides [7]. The major adverse events associated with treatment with colistin include nephrotoxicity and neurotoxicity [8]. Concerning these toxicities, the necessity of administering a loading dose of colistin is debated in clinical practice. With regard to the therapeutic efficacy, the administration of a loading dose is suggested because the plasma concentration of colistin increases slowly over hours or even days to reach the ideal level, and a better clinical cure rate and microbiological outcome were reported in a specific population after the administration of a loading dose [9, 10]. Regarding nephrotoxicity, the risk of developing acute kidney injury (AKI) after a loading dose of colistin is administered is unclear. Some studies showed a significant correlation between the administration of a loading dose and nephrotoxicity, while other studies reported that renal impairment could be prevented by some measures, such as avoiding the concomitant prescription of nephrotoxic medicines and treatment of the patient in the intensive care unit (ICU) [11–13]. The optimal method of colistin administration to maximize the therapeutic efficacy and minimize the risk of renal injury needs to be verified in more studies.

We reviewed five retrospective studies published in recent years [13–17], and observed approximate 26–52% of patients did not receive the administration of loading dose when intravenous colistin was prescribed for treatment of CRGNB associated infection, implying the importance or necessity of loading dose has not been well established in clinical practice. Furthermore, international consensus guidelines recommend the prescription of a loading dose when initiating intravenous colistin therapy but emphasize that more evidence is needed regarding the efficacy and safety of the administration of a loading dose [18]. In the present study, we constructed a multicenter, retrospective cohort study to investigate the impact of the administration of a loading dose of colistin on the clinical and microbiological outcomes and AKI in patients with CRGNB-associated HAP/VAP who were treated in the ICU.

## Methods

### Study population and data collection

**Fig. 1:**
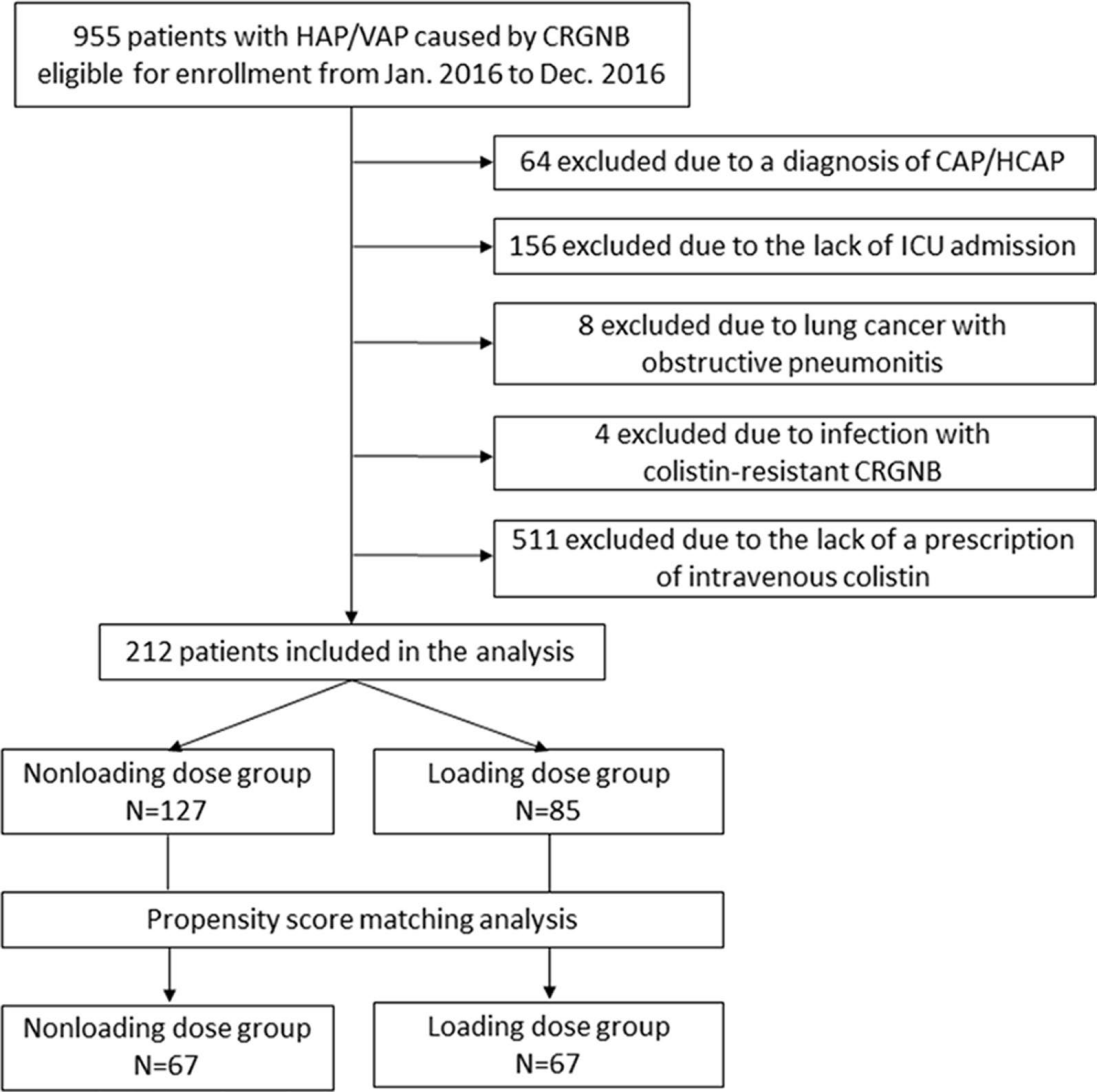
Flow diagram of patient inclusion and exclusion

This retrospective study was conducted in five medical centers in Taiwan and recruited ICU-admitted patients who had colistin-susceptible CRGNB-associated pneumonia from January 2016 to December 2016. Associated studies have been in preparation or published [19, 20]. The flow diagram of this article for patient inclusion and exclusion is shown in Fig. 1. The pneumonia index date (pneumonia onset day) was defined as the date of specimen collection. The inclusion criteria included (A) ICU-admitted patients who were diagnosed with nosocomial pneumonia that developed more than 48 h after admission and (B) the growth of CRGNB from respiratory specimens that was resistant to at least one kind of tested carbapenems. The exclusion criteria included age younger than 20 years, community-acquired pneumonia or healthcare-associated pneumonia, concomitant lung cancer with obstructive pneumonitis, CRGNB that were resistant to colistin, and no intravenous colistin prescribed within 7 days of the index date for pneumonia.

The demographic characteristics and baseline variables were retrieved from the medical records. The assessment of disease severity was made by calculating the Acute Physiology and Chronic Health Evaluation (APACHE) II score on the day of ICU admission and the Sequential Organ Failure Assessment (SOFA) score on the day of ICU admission and pneumonia index date. We also collected other variables associated with organ dysfunction, including septic shock, mechanical ventilator use, the PaO2/FiO2 (P/F) ratio, and renal replacement therapy, on the pneumonia index date.

### Nosocomial pneumonia and microbiological tests

The diagnosis of pneumonia was based on new or progressive infiltration on chest radiography accompanied by at least two clinical findings, including cough, purulent sputum production, fever (> 38 °C) or hypothermia (< 36 °C), leukocytosis (plasma white cell count > 10,000 per mm^3^), leukopenia (plasma white cell count < 4000 per mm^3^) or band cell percentage > 10%. Eligible specimens were collected from sputum, tracheal aspirates, or bronchoalveolar lavage fluid with a CRGNB concentration greater than 10^4^ colony forming units per ml. The pneumonia index date (pneumonia onset day) was defined as the date of specimen collection. The determination of susceptibility to carbapenems of the causative GNB was performed according to the Clinical and Laboratory Standards Institute recommendations.

### Colistin loading dose and therapeutic regimens

All the patients in this study were treated with intravenous colistimethate sodium, and we classified these patients into colistin loading dose and colistin nonloading dose groups. The administration of a loading dose of intravenous colistin was defined as the achievement of colistin base activity (CBA) = an average steady-state plasma concentration of colistin (C_ss,avg_) target (mg/L) × 2.0 × ideal body weight (kg); the target C_ss,avg_ was 2 mg/L, and the maximum loading dose was 300 mg of CBA [21]. Patients who were administered a loading dose in accordance with the above definition were classified in the loading dose group, while the other patients who received either no loading dose or an inadequate loading dose were classified in the nonloading dose group. The daily dose of intravenous colistin in both groups was prescribed according to the recommendations [22]. Antibiotics, including colistin (intravenous and inhaled), sulbactam, carbapenem, and tigecycline, that were administered for 2 or more days were recorded in this study.

### Outcomes and nephrotoxicity evaluations

The primary outcomes of this study were the mortality rate, clinical response, and microbiological response at days 7, 14 and 28. The clinical response to treatment was classified as a cure (resolution of symptoms and freedom from antibiotics), improvement (partial resolution of symptoms but still needing treatment with antibiotics) and failure (no resolution of symptoms or death). Clinically favorable outcomes were defined as both cure and improvement. The microbiological response to treatment was classified as eradication (no growth of causative pathogens in at least two consecutive respiratory specimens), persistence (persistent growth of causative pathogens in respiratory specimens), recurrence (reisolation of causative pathogens within 14 days of eradication), and undetermined (follow-up specimen unavailable or only one specimen with no growth). The microbiological eradication rate was defined as the ratio of the number of cases of eradiation to the sum of the number of cases of eradiation, persistence and recurrence (not including undetermined).

The secondary outcomes included the length of hospital stay, the length of ICU stay, 28-day ventilator weaning rate, and nephrotoxicity. The assessment of hospital and ICU stays did not include patients who died during hospitalization. We evaluated nephrotoxicity based on the development of acute kidney injury (AKI), which was defined according to the Kidney Disease Improving Global Outcomes (KDIGO) criteria (creatinine increase ≥ 0.3 mg/dL within 2 days or ≥ 50% from baseline within 7 days) [23]. The analysis of AKI did not include patients who were receiving renal replacement therapy at baseline or had insufficient creatinine data to enable the assessment of AKI.

### Propensity-score matching analysis

Due to differences in demographic characteristics and disease severity between the loading dose and nonloading dose groups, we performed a propensity-score matching (PS matching) analysis with 1:1 matching and a 0.2 caliper width to investigate the outcomes. The PSs were calculated by the logistic regression of variables including age, sex, pathogen, pneumonia type, ICU type, coadministered antibiotics (carbapenem, tigecycline), comorbidities (lung cancer, malignancy, liver disease, heart failure, hypertension, stroke, degenerative brain diseases, lung diseases, diabetes, autoimmune diseases), and SOFA score on the pneumonia index date.

### Statistical analysis

**Fig. 2:**
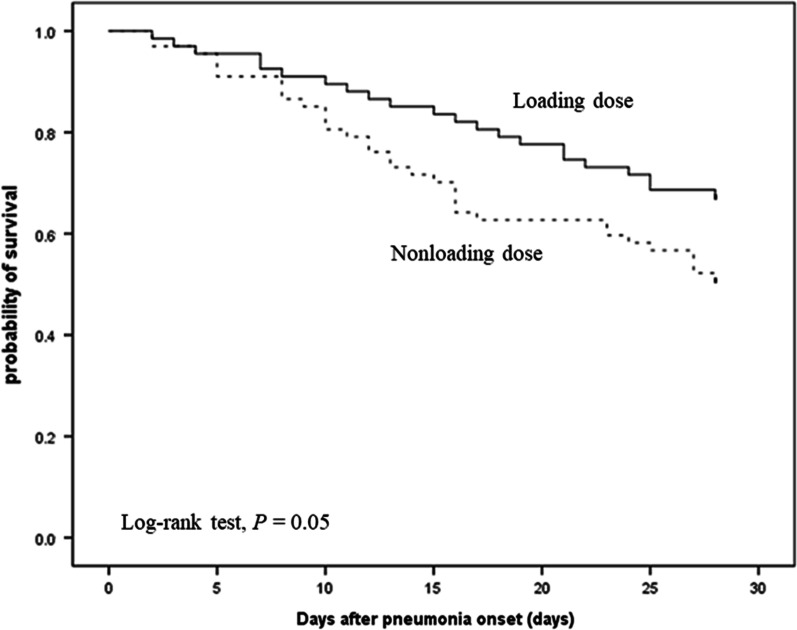
Kaplan–Meier analysis of survival in patients treated with a loading dose or a nonloading dose of intravenous colistin after propensity score matching

Continuous variables are expressed as the means ± standard deviations, and categorical variables are expressed as percentages. The differences in continuous and categorical variables were compared with the Mann–Whitney U test, Chi-square test, or Fisher’s exact test in Tables 1, 2 and 3. After PS matching, there was no significant difference of demographic characteristics and disease severities between loading and nonloading dose group by univariate analysis in Table 2. Thus, we just added age and gender for multivariate analysis of clinical factors associated with treatment outcomes after PS matching in Table 4. The Cox proportional hazards model was used to estimate the hazard ratios and 95% confidence intervals for 28-day all-cause mortality; the logistic regression analysis was used to estimate the odds ratios and 95% confidence intervals for favorable clinical outcomes and microbiological eradication at day 14. A subgroup analysis was performed to evaluate the therapeutic benefits of the administration of a loading dose in each subgroup in Supplementary Figure S1. Kaplan–Meier analysis and log-rank tests were used to compare survival between the loading dose and nonloading dose groups in Fig. 2. The statistical analyses were performed with SPSS software version 18.0 (SPSS Inc., Chicago, IL). A *P* value ≤ 0.5 was considered statistically significant. This study was approved by the Institutional Review Boards of all the participating hospitals (registration numbers: 2018-03-001CC, 1-107-05-054, CE18100A, CMUH107-REC3-052, and KMUHIRB-E(I)-20180141).

**Table 1:** Demographic characteristics and disease severities of ICU patients treated with nonloading or loading colistin

	Nonloading dose (*n* = 127)	Loading dose (*n* = 85)	*P* value
Age, M (SD)	69.67 (15.73)	69.42 (14.50)	0.909
Sex, *n* (%)			0.823
Female	51 (40.2)	32 (37.6)	
Male	76 (59.8)	53 (62.4)	
Height, M (SD)	161.97 (10.14)	161.49 (7.94)	0.717
Weigh, M (SD)	60.08 (14.54)	60.13 (15.80)	0.983
BMI, M (SD)	22.55 (5.03)	23.10 (5.70)	0.479
Smoking	44 (35.2)	32 (37.6)	0.829
Alcohol consumption	23 (18.4)	15 (17.9)	1.000
Pathogen, *n* (%)			0.052
CR-Pseudo	8 (6.3)	8 (9.4)	
CRAB	115 (90.6)	68 (80.0)	
CRKP	4 (3.1)	9 (10.6)	
Pneumonia types, *n* (%)			0.001
HAP	27 (21.3)	38 (44.7)	
VAP	100 (78.7)	47 (55.3)	
ICU types, *n* (%)			0.063
Medical ICU	89 (70.1)	70 (82.4)	
Surgical ICU	38 (29.9)	15 (17.6)	
Comorbidities			
Lung cancer, *n* (%)	8 (6.3)	2 (2.4)	0.322
Malignancy	17 (13.4)	9 (10.6)	0.693
Liver disease	14 (11.0)	13 (15.3)	0.481
Heart failure	14 (11.0)	11 (12.9)	0.836
Hypertension	69 (54.3)	40 (47.1)	0.369
Stroke	20 (15.7)	12 (14.1)	0.897
Degenerative brain disease	16 (12.6)	8 (9.4)	0.620
Renal insufficiency	17 (17.0)	14 (29.2)	0.137
Lung disease	22 (17.3)	26 (30.6)	0.036
Diabetes	43 (33.9)	33 (38.8)	0.553
Autoimmune disease	11 (8.7)	6 (7.1)	0.870
Coadministered antibiotics			
Sulbactam, *n* (%)	6 (4.7)	1 (1.2)	0.247
Carbapenem	60 (47.2)	27 (31.8)	0.035
Tigecycline	40 (31.5)	42 (49.4)	0.013
Inhaled colistin	50 (39.4)	35 (41.2)	0.904
Disease severity			
APACHE II score, M (SD)	22.30 (8.30)	23.86 (8.09)	0.187
SOFA score (ICU admission date), M (SD)	7.80 (3.83)	9.71 (3.68)	< 0.001
SOFA score (pneumonia index date), M (SD)	8.04 (3.56)	9.35 (3.65)	0.010
Septic shock	21 (16.5)	27 (31.8)	0.015
Invasive ventilator	109 (85.8)	79 (92.9)	0.167
PF ratio, M (SD)	269.21 (120.24)	255.27 (139.94)	0.462
Dialysis (HD + CVVH)	21 (16.5)	14 (16.5)	1.000
Lab data analysis			
Leukocyte, M (SD)	13,441.97 (8020.94)	13,968.54 (9484.37)	0.664
C-reactive protein, M (SD)	13.47 (21.66)	11.94 (8.96)	0.557
Albumin, M (SD)	2.63 (0.56)	2.55 (0.48)	0.280
Creatinine, M (SD)	2.07 (1.78)	2.14 (2.06)	0.773

M (SD): Mean (standard deviation)

**Table 2:** Demographic characteristics and disease severities of ICU patients treated with a nonloading dose or loading dose of colistin after propensity-score matching

	Nonloading dose (*n* = 67)	Loading dose (*n* = 67)	*P* value
Age, M (SD)	68.79 (16.83)	69.78 (14.66)	0.718
Sex, *n* (%)			1.000
Female	29 (43.3)	29 (43.3)	
Male	38 (56.7)	38 (56.7)	
Height, M (SD)	160.17 (10.44)	161.83 (7.92)	0.326
Weigh, M (SD)	58.8 (15.61)	60.33 (15.31)	0.580
BMI, M (SD)	22.38 (6.03)	23.00 (5.45)	0.553
Smoking	24 (36.4)	23 (34.3)	0.949
Alcohol consumption	10 (14.9)	10 (15.2)	1.000
Pathogen, *n* (%)			0.867
CR-Pseudo	5 (7.5)	6 (9.0)	
CRAB	59 (88.1)	57 (85.1)	
CRKP	3 (4.5)	4 (6.0)	
Pneumonia types, *n* (%)			0.464
HAP	20 (29.9)	25 (37.3)	
VAP	47 (70.1)	42 (62.7)	
ICU types, *n* (%)			0.827
Medical ICU	55 (82.1)	53 (79.1)	
Surgical ICU	12 (17.9)	14 (20.9)	
Comorbidities			
Lung cancer, *n* (%)	1 (1.5)	2 (3.0)	1.000
Malignancy	8 (11.9)	8 (11.9)	1.000
Liver disease	7 (10.4)	8 (11.9)	1.000
Heart failure	9 (13.4)	8 (11.9)	1.000
Hypertension	34 (50.7)	35 (52.2)	1.000
Stroke	10 (14.9)	11 (16.4)	1.000
Degenerative brain disease	9 (13.4)	7 (10.4)	0.790
Renal insufficiency	7 (14.9)	12 (27.9)	0.210
Lung diseases	19 (28.4)	17 (25.4)	0.845
Diabetes	28 (41.8)	27 (40.3)	1.000
Autoimmune disease	5 (7.5)	4 (6.0)	1.000
Coadministered antibiotics			
Sulbactam, *n* (%)	2 (3.0)	1 (1.5)	1.000
Carbapenem	27 (40.3)	24 (35.8)	0.722
Tigecycline	32 (47.8)	30 (44.8)	0.862
Inhaled colistin	27 (40.3)	27 (40.3)	1.000
Disease severity			
APACHE II score, M (SD)	22.29 (8.38)	23.37 (8.33)	0.464
SOFA score (ICU admission date), M (SD)	8.54 (3.63)	9.39 (3.81)	0.188
SOFA score (pneumonia index date), M (SD)	8.46 (3.69)	8.63 (3.36)	0.788
Septic shock	12 (17.9)	18 (26.9)	0.300
Invasive ventilation	58 (86.6)	61 (91.0)	0.584
PF ratio, M (SD)	261.24 (121.82)	249.85 (134.43)	0.623
Dialysis (HD + CVVH)	13 (19.4)	8 (11.9)	0.342
Lab data analysis			
Leukocyte, M (SD)	13,402.39 (8335.61)	13,190.24 (8479.92)	0.884
C-reactive protein, M (SD)	14.56 (28.26)	11.31 (8.62)	0.396
Albumin, M (SD)	2.57 (0.57)	2.58 (0.51)	0.852
Creatinine, M (SD)	1.97 (1.88)	2.07 (2.03)	0.755

M (SD): Mean (standard deviation)

**Table 3:** Therapeutic efficacy and acute kidney injury in the loading dose and nonloading dose groups after propensity score matching

	Nonloading dose (*n* = 67)	Loading dose (*n* = 67)	*P* value
Length of hospital stay (days), M (R)	60 (20–220)	52 (14–284)	0.037^a^
Length of ICU stay (days), M (R)	22 (3–215)	20 (7–95)	0.765^a^
28-day ventilator weaning	34 (53.1)	29 (44.6)	0.429
Mortality (since pneumonia onset)			
Day 7, *n* (%)	6 (9.0)	5 (7.5)	1.000
Day 14, *n* (%)	19 (28.4)	10 (14.9)	0.093
Day 28, *n* (%)	33 (49.3)	22 (32.8)	0.079
In-hospital mortality, *n* (%)	42 (62.7)	32 (47.8)	0.118
Favorable clinical outcomes			
Day 7	23 (49.3)	39 (58.2)	0.386
Day 14	24 (35.8)	37 (55.2)	0.037
Day 28	26 (38.8)	37 (55.2)	0.083
Microbiological eradication			
Day 7	2 (5.0)	7 (20.0)	0.101
Day 14	12 (27.3)	19 (50.0)	0.042
Day 28	19 (45.2)	26 (60.5)	0.234
Acute kidney injury	27 (50.0)	31 (55.4)	0.710

M (R): Median (range); ^a^ Mann–Whitney U test; MV: Mechanical ventilation

The assessment of hospital and ICU stays did not include patients who died during hospitalization

Definition of acute kidney injury: creatinine increase ≥ 0.3 mg/dL within 2 days or ≥ 50% from baseline within 7 days according to the KDIGO criteria; The comparison of AKI did not include the patients who were receiving renal replacement therapy at baseline and those who lacked adequate creatinine data for the assessment of AKI

**Table 4:** Multivariate analysis of clinical factors associated with treatment outcomes after propensity score matching

	28-Day all-cause mortality^a^			Favorable clinical outcomes on day 14^b^		Microbiological eradication day 14^b^	
	aHR (95% CI)		*P* value	aOR (95% CI)	*P* value	aOR (95% CI)	*P* value
Loading dose	0.59 (0.34–1.01)		0.054	2.24 (1.12–4.52)	0.024	2.80 (1.10–7.12)	0.031
Age	1.01 (0.99–1.02)		0.594	1.00 (0.97–1.02)	0.650	1.01 (0.99–1.04)	0.334
Male	1.35 (0.77–2.35)		0.291	1.51 (0.74–3.09)	0.255	1.23 (0.49–3.13)	0.659

^a^Adjusted hazard ratio (aHR) and 95% confidence interval (CI) were derived from Cox regression analysis

^b^Adjusted odds ratios (aORs) and 95% CIs were derived from logistic regression analysis

## Results

### Demographic characteristics and disease severities

The comparison of demographic characteristics of the loading dose and nonloading dose groups is shown in Table 1. The nonloading dose group had a significantly higher proportion of patients who were diagnosed with VAP than the loading dose group (*p* = 0.001). There were no significant differences in comorbidities between the two groups except lung diseases, including asthma, chronic obstructive pulmonary disease, interstitial lung disease, bronchiectasis, and active tuberculosis, which were significantly more common in the loading dose group (17.3% vs. 30.6%, *p* = 0.036). Regarding coadministered antibiotics, the nonloading dose group had a significantly higher proportion of patients with the concurrent administration of carbapenem in addition to intravenous colistin.

As for disease severity, the loading dose group had significantly more severe disease than the nonloading dose group according to the SOFA score on ICU admission (9.71 vs. 7.80, *p* < 0.001), pneumonia index date (9.35 vs. 8.04, *p* = 0.010), and proportion of patients with septic shock (31.8% vs. 16.5%, *p* = 0.015).

### Therapeutic efficacy after PS matching

In Table 2, we conducted PS matching analysis before analyzing the primary and secondary outcomes, and there were no significant differences in baseline demographic characteristics and disease severities between nonloading (*n* = 67) and loading groups (*n* = 67). Table 3 shows the loading dose group had a significantly higher proportion of patients with clinically favorable outcomes (55.2% vs. 35.8%, *p* = 0.037) and microbiological eradication (50.0% vs. 27.3%, *p* = 0.042) at day 14 than the nonloading dose group. With regard to all-cause mortality, the mortality rates were not significantly different (but favor the loading dose group) at days 7, 14, 28 or throughout hospitalization. However, the Kaplan–Meier analysis of 28-day survival showed that the loading dose group had a significantly longer survival duration than the nonloading dose group (log rank test = 0.05) (Fig. 2). Table 4 shows the administration of a loading dose is an independent factor for favorable clinical outcomes and microbiological eradication on day 14. The evaluation of the secondary outcomes showed that the loading dose group had a significantly shorter length of hospital stay than the nonloading dose group (52 vs. 60, *p* = 0.037).

For evaluating the therapeutic benefits of the administration of a loading dose compared to nonloading dose in each subgroup, subgroup analysis was performed in Additional file 1: Fig. S1. We observed that the subgroup with a PF ratio ≤ 235 had relatively better primary outcomes, including 28-day all-cause mortality and clinically favorable outcomes and microbiological eradiation on day 14, than those with a PF ratio > 235.

### Nephrotoxicity after PS matching

We compared the development of AKI after the administration of intravenous colistin in the loading dose and nonloading dose groups in Table 3. There was no significant difference in the risk of developing AKI between the groups.

## Discussion

This multicenter, retrospective cohort study demonstrated that the loading dose group had a shorter length of hospital stay, better clinical and microbiological outcomes on day 14, and longer survival (KM analysis) than the nonloading dose group. With regard to nephrotoxicity, the loading dose group did not have a higher risk of developing AKI than the nonloading dose group.

A large prospective cohort conducted by Katip et al. [24] recruited patients in the general ward and ICU with MDR *A. baumannii* infection and showed a significantly higher microbiological eradiation rate in the colistin loading dose group than in the nonloading dose group, while other retrospective studies showed that there was no significant difference in microbiological eradiation rates between the two groups [15, 17]. This disparity is attributable to the different research designs, causative pathogens, and levels of disease severity between studies. Our study demonstrated that the loading dose group had a significantly higher microbiological eradication rate than the nonloading dose group, and the colistin loading dose strategy was an independent factor affecting microbiological eradication at day 14. This trend was also observed at day 7 and day 28. In addition, one recent meta-analysis reported that the clinical cure rate was similar between the loading dose and nonloading dose groups [25]. Our study further demonstrated that the loading dose group had a significantly higher possibility of clinically favorable outcomes than the nonloading dose group at day 14, although this therapeutic benefit was less pronounced at day 7 and day 28. Furthermore, our study showed that there was no significant difference (but favor the loading dose group) in the mortality rate between the groups throughout hospitalization or on days 7, 14 and 28, which was consistent with the findings of other studies [15, 17, 24]. It is interesting and worth mentioning that the present study demonstrated that the loading dose group had significantly longer survival than the nonloading dose group according to the Kaplan–Meier analysis (Fig. 2). Hence, the survival benefit of the loading strategy needs to be clarified in future studies.

Nephrotoxicity is a major adverse effect of colistin, and pharmacokinetic studies have reported that a C_ss,avg_ of colistin > 2.5 mg/L increased the risk of nephrotoxicity [26, 27], which could be a result of the administration of loading dose, that led to the fluctuation in the level of C_ss,avg_. A meta-analysis reported that there was no difference in the risk of AKI between the loading dose and nonloading dose groups, but the outcomes and the definition of AKI in each study included in the analysis were clearly different [25]. For example, Katip and Jung applied the RIFLE and AKIN criteria, respectively, to define AKI and observed that the risk of AKI was similar in the loading dose and nonloading dose groups [16, 24], while Rigatto and Shields used the RIFLE and KDIGO criteria, respectively, and found that the risk of AKI was significantly higher in the loading dose group than in the nonloading dose group [11, 13]. The present study showed that there was no significant difference in the risk of developing AKI between the groups based on the KDIGO criteria after PS matching of important covariates. Although the therapeutic benefit of a loading dose of colistin may justify the potential risk of AKI, as suggested by the guidelines [18], our findings provide further evidence of its safety, reassuring clinicians concerned about kidney injury in critically ill and vulnerable patients.

There were some strengths of the current study. First, this article is the first to adopt PS matching to analyze the therapeutic benefit and risk of AKI associated with the administration of colistin, with or without a loading dose. This strategy minimized the differences in baseline characteristics between groups, such as disease severity and comorbidities, that could have seriously affected the outcomes. Second, subgroup analysis was applied to investigate which subgroup received the maximum benefit from the loading dose strategy, and we identified that the loading dose group with a PF ratio ≤ 235 experienced the greatest therapeutic benefit, including a lower day 28-day all-cause mortality and better clinical and microbiological outcomes on day 14. These findings provide clinicians with evidence that the administration of a loading dose is warranted, especially when prescribing intravenous colistin to critically ill patients with a low PF ratio. Third, this is a multicenter study, which could decrease the possibility of selection bias, and took different settings of clinical practice into account. However, there were some limitations of this study. First, there were only 67 patients in each group after PS matching, so other therapeutic benefits (Table 3) of the loading dose strategy may not have been observed due to the limited statistical power, although it was sufficient to demonstrate the superior therapeutic benefit of a loading dose compared to a nonloading dose. Second, we only enrolled patients with carbapenem-resistant pathogens, so the effectiveness of the loading dose strategy for other pathogens needs further investigation. Third, all the patients recruited for this study were treated in the ICU, so the findings cannot be extrapolated to other clinical settings.

## Conclusions

This study demonstrated that the administration of a loading dose of intravenous colistin yielded multiple therapeutic benefits in ICU patients with nosocomial pneumonia caused by CRGNB, and we did not observe a difference in the risk of developing AKI compared to the nonloading. Our study provides more evidence to strengthen the necessity and confidence in the efficacy and safety of the administration of a loading dose of intravenous colistin.

## Supplementary Information

**Additional file 1: Figure S1**. Subgroup analysis to evaluate the therapeutic benefits of the administration of a loading dose in each subgroup after propensity score matching

## Data Availability

The datasets used and/or analyzed during the current study are available from the corresponding author on reasonable request.
