# Supplementary figures and images for: The necessity of a loading dose when prescribing intravenous colistin in critically ill patients with CRGNB-associated pneumonia: a multi-center observational study
Source: Crit Care. 2022 Apr 4;26:91. doi: 10.1186/s13054-022-03947-9 (PMC8981852; doi:10.1186/s13054-022-03947-9)

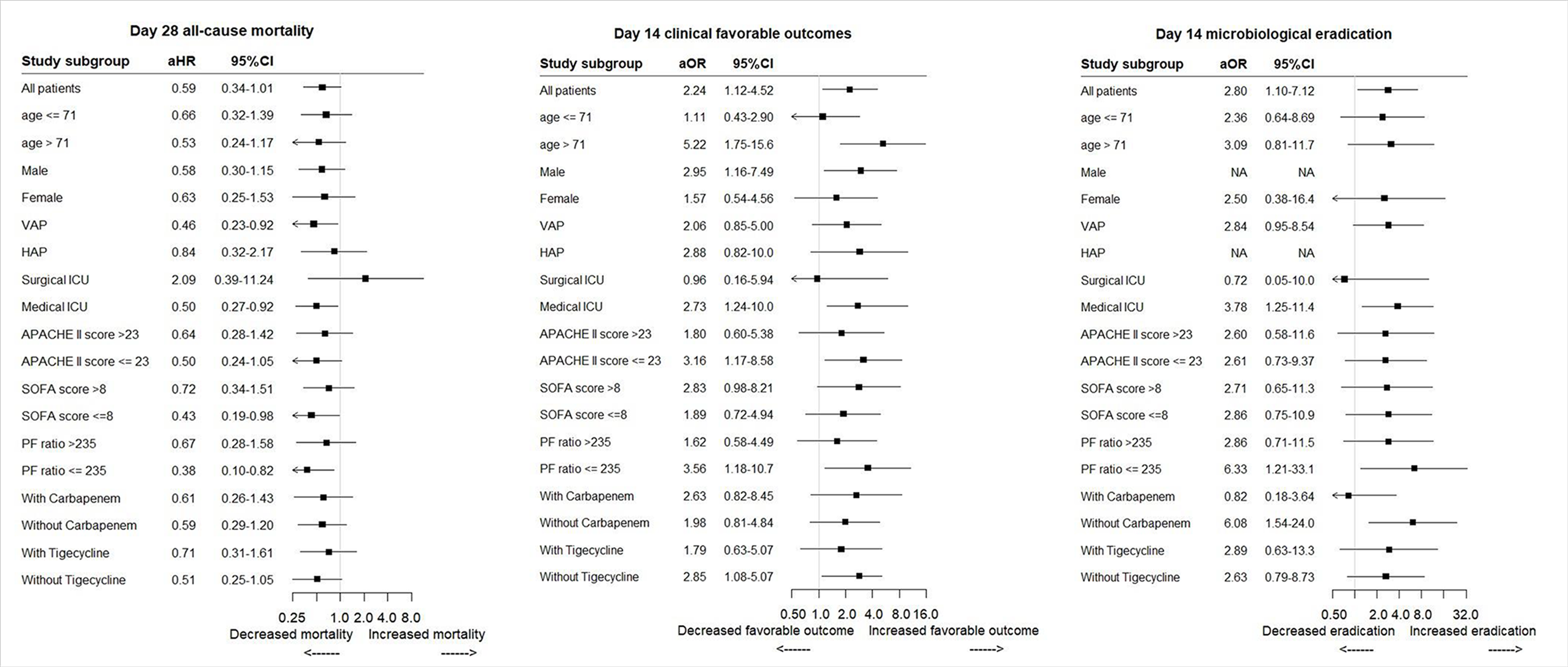

Supplement: Supplementary file 1 — Additional file 1: Figure S1. Subgroup analysis to evaluate the therapeutic benefits of the administration of a loading dose in each subgroup after propensity score matching [file 13054_2022_3947_MOESM1_ESM.tif]
